# Does Village Water Supply Affect Children’s Length of Stay in a Therapeutic Feeding Program in Niger? Lessons from a Médecins Sans Frontières Program

**DOI:** 10.1371/journal.pone.0050982

**Published:** 2012-12-07

**Authors:** Claire Dorion, Paul R. Hunter, Rafael Van den Bergh, Carme Roure, Pascale Delchevalerie, Tony Reid, Peter Maes

**Affiliations:** 1 Médecins Sans Frontières, Operational Center Barcelona, Barcelona, Spain; 2 The Norwich School of Medicine, University of East Anglia, Norwich, United Kingdom; 3 Médecins Sans Frontières, Operational Center Brussels, Brussels, Belgium; UCL Institute of Child Health, University College London, United Kingdom

## Abstract

**Objective:**

With an increasing move towards outpatient therapeutic feeding for moderately and severely malnourished children, the home environment has become an increasingly important factor in achieving good program outcomes. Infections, including those water-borne, may significantly delay weight gain in a therapeutic feeding program. This study examined the relationship between adequacy of water supply and children’s length of stay in a therapeutic feeding program in Niger.

**Methods:**

The length of stay in a therapeutic feeding program of Médecins Sans Frontières in Niger was registered for 1518 children from 20 villages in the region. In parallel, the quality and quantity of the water source in each village were documented, and the association between adequacy of the water supply and length of stay in the program was assessed through Generalized Estimating Equation analysis.

**Results:**

36% of the children presented with a secondary infection, 69% of which were water-related. When stratified by the adequacy of the quantity and/or quality of the water supply in their village of origin, non-adequacy of the water supply was clearly associated with a higher prevalence of secondary water-related infections and with much longer lengths of stay of malnourished children in the therapeutic feeding program.

**Conclusion:**

This study suggests that therapeutic feeding programs using an outpatient model should routinely evaluate the water supply in their target children’s villages if they are to provide optimal care.

## Introduction

Over the past few years there has been a dramatic shift in strategy for managing malnutrition in low income countries. Rather than admission to therapeutic feeding centers, where they receive milk-based feedings, ambulatory treatment of children with moderate and severe malnutrition using Ready To Use Therapeutic Foods (RUTF) such as Plumpy Nut is increasingly recommended, in order to improve program coverage and acceptability and to reduce program costs and facility-associated complications such as nosocomial infections[1]–[2].

The shift from inpatient to outpatient care changes the treatment context from one where food, medicines, soap and water are provided in a controlled environment to one where contact with patients is intermittent. Although food, medicines and soap are typically still provided for the outpatients, control or monitoring of their correct use is not possible. Additionally, there is no programmatic control over the water supply of the children in care.

There is some literature regarding the relationship between malnutrition and infection[1], recently reviewed in [2]. Numerous studies have reported on the vicious cycle of nutrient deficiency adversely affecting immunocompetence, leading to greater sensitivity to opportunistic infections, and infections reducing appetite and nutrient adsorption capacity[1], [3], [4]. More recent research has highlighted the interaction between occurrence of diarrheal diseases and malnutrition[3], [5], [6]. A predictive model, based on published studies in Colombia, Guatemala and Peru, clearly demonstrates that the energy intake required to achieve a certain nutritional status is increased in children with diarrhea[7]. Unless the infection is addressed, a much longer time is required to achieve adequate nutritional status. A vital component in preventing diarrheal disease and other infections is safe water; however, ensuring this in ambulatory program settings is a challenge [8].

**Figure 1:**
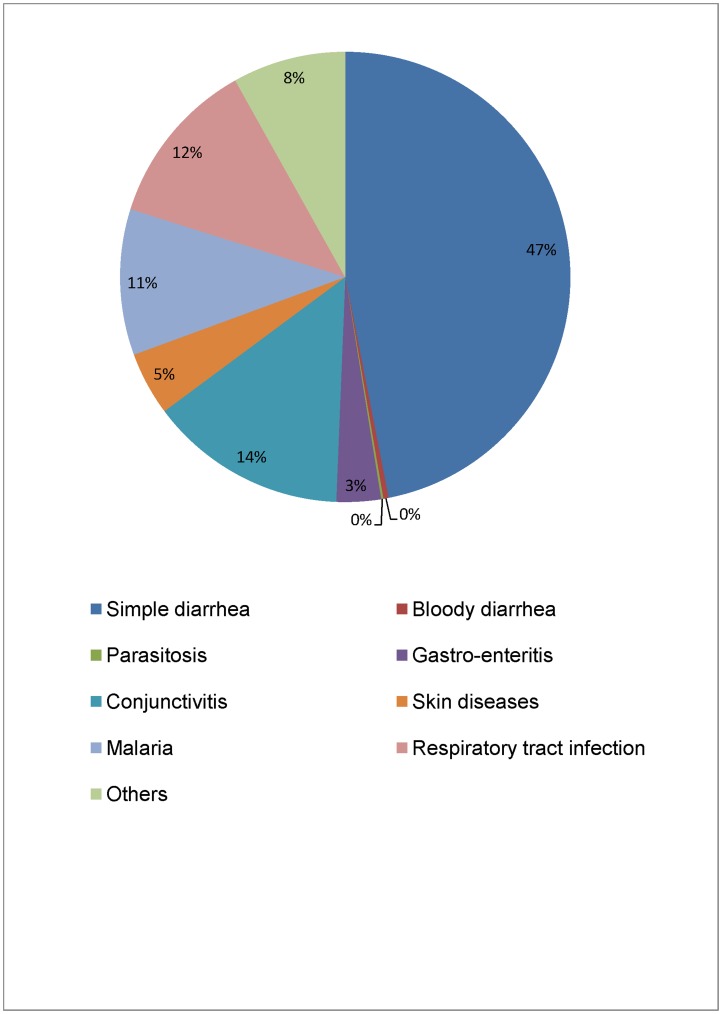
Proportion of secondary infections among children from the 20 selected study villages presenting for primary care.

The recommendations on shifting to outpatient care were implemented in a Médecins Sans Frontières (MSF) nutrition program in the Tahoua region in Niger, where children with moderate acute malnutrition have been treated since 2005. Mortality and nutrition surveys conducted internally by MSF in the region indicated levels of global acute malnutrition ranging 7.8–10.3%, and severe acute malnutrition levels ranging 0.3–1.0%. The ambulatory program operated from five nutritional centers, covering a total of 70 villages. Moderately acutely malnourished children received RUTF support, and were only discharged from the program if they fulfilled the discharge criteria of >−2 weight-for-height Z-scores on two consecutive weight measurements, absence of oedema for at least one week, absence of acute medical problems, and a mid-upper arm circumference > 115 mm.

In 2007, this program performed an internal assessment and discovered that children were remaining in care much longer than expected (i.e. more than the six weeks observed in similar programs[9], [10]). Since this program operated on an outpatient basis, the hypothesis that problems with water might be partly responsible was proposed. A literature search on PubMed and Google Scholar did not find any studies demonstrating a link between water quality and outcomes of ambulatory therapeutic feeding programs.

Based on this concern, a formal study was proposed to assess the putative association between adequacy of water supplies and length of stay in an outpatient therapeutic feeding program in the Tahoua region of Niger.

## Methods

### Design

This was an analysis of routinely-collected program and observational data.

**Figure 2:**
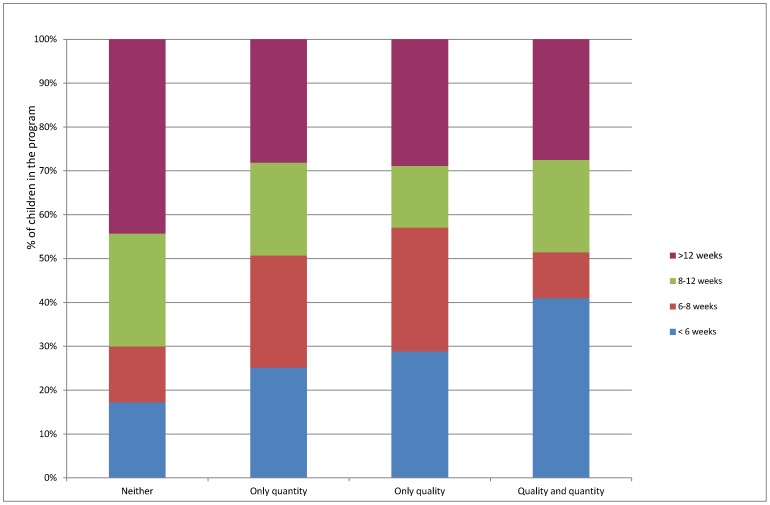
Comparison of the length of stay in an MSF feeding program versus adequacy of the village water supply.

### Setting

The study was carried out in the five MSF nutritional centers – Madaoua, Aouloumatt, Saban Guidan, Manzou and Bangui – in the Tahoua region in southern Niger. These centers have treated approximately 7000 children from an under-fives population of 21,000 since 2005[11].

### Sample

Out of 70 villages in the region, we chose to examine those with at least 30 children receiving therapeutic feeding and those with the highest burden of secondary infections. Twenty villages with these characteristics were randomly selected by allocating random numbers generated in Microsoft Excel to them and selecting the top 20. These villages represented about 40% of all children in the feeding programs at the time of the study. All moderately malnourished children from these villages were included in this analysis, while severely malnourished children were excluded: severely malnourished children represented too small a group for reliable statistical analysis, and rarely stayed in ambulatory care for continuous stretches of time due to frequent admissions to inpatient centers, further complicating the analysis.

**Figure 3:**
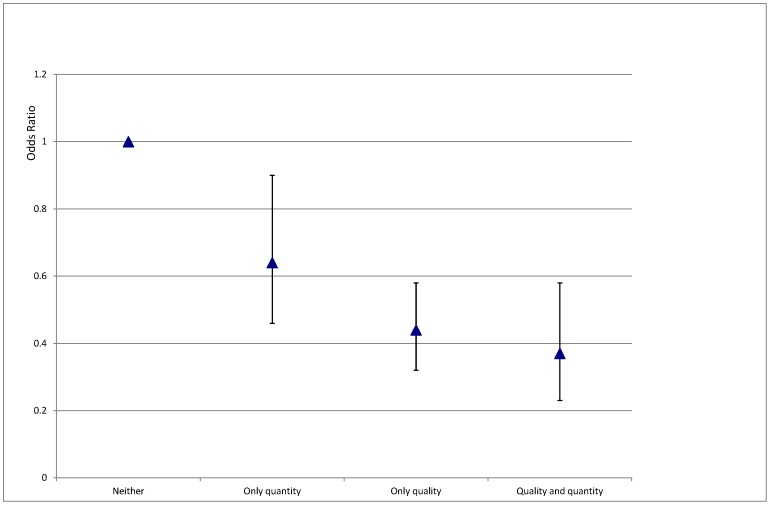
Odds ratio’s for a shorter length of stay, as assessed by ordinal logistic regression (odds of an individual with a shorter length of stay falling in a category with less adequacy of water supply).

### Outcomes

The primary outcome was the length of stay in the therapeutic feeding program: the time from registration till formal discharge.

### Variables

Moderately malnourished children were defined as those whose weights were between 70% and 80% of median weight for height using the National Center for Health Statistics (NCHS) reference.

Adequacy of the village water supply was stratified in two categories and defined as follows:

Adequate water quality was defined as all groundwater from a protected source – e.g. a borehole with protective apron and functional hand or submersible pump. A village was only classified as having adequate water quality if all water sources used for human consumption could be considered adequate. Surface water or water provided by open wells was considered of inadequate quality. As such, our definition represents a proxy indicator for true water quality and is in accordance with definitions used previously by the WHO and UNICEF[12].
Adequate water quantity was defined as a capacity of the water source of at least 20 L per person per day.

Secondary infections were defined as:

Diarrhea with or without blood; at least three episodes per day
Parasitosis: any parasite infection
Gastroenteritis: inflammation of the gastrointestinal tract that caused nausea and vomiting as well as diarrhea
Skin infections: including scabies and impetigo
Conjunctivitis: any acute inflammation of the conjunctiva
Malaria: symptoms (fever, headache, vomiting) with positive Paracheck
Respiratory tract infection: any combination of cough, fever, sputum and wheezing
Others: infections that did not fit any of the above definitions

The first five of these conditions (diarrhea, parasitosis, gastro-enteritis, skin infections and conjunctivitis) were considered water-related infections.

### Study Procedure

Data were collected from three sources. The first was the medical register book from nutritional centers, which contained as variables the name, date of admission, village of origin, secondary infections, nutritional status (weight for height) and date of the discharge of the patients.

As part of routine program evaluation, field visits to the selected villages provided the second source of data. After obtaining consent from the village leaders, all water sources were examined.

Thirdly, geographic locations (GPS positions) of the selected villages and the MSF nutritional centers were obtained from Google Earth® and distances between the villages and their respective closest nutritional center were calculated using the haversine formula[13].

We used patient data aggregated across six months of the year: three months during the dry season and three during the rainy season, to compensate for possible differential variation of the water quality versus quantity in the dry versus the rainy season.

### Analysis

Data from the medical registers were entered into an Excel spreadsheet and STATA version 10 software (Stata corporation, Texas, USA) was used for analysis. To test the association between length of stay and village water quality/quantity, Generalized Estimating Equation analysis was performed using an ordinal logistic regression model. In this model, length of stay was allocated into one of 4 categories: <6 weeks, 6 to <8 weeks, 8 to <12 weeks and ≥12 weeks.

### Ethics

The study met the MSF Ethics Review Board’s approved criteria for analysis of routinely-collected program data. Additionally, the study was approved by the *Directeur du bureau hydraulique de la region de Tahoua* in Niger. Analysis was done on previously collected program data only: patients were not contacted, and no identifying characteristics of patients were collected. As such, informed consent of involved patients was not indicated or sought.

## Results

Across the 20 selected villages, 3618 children presented for primary care during the study period. Among these children, 1302 (36%) presented with a secondary infection at the time of admission. Water-related infections, such as feco-orally transmitted diseases (diarrhea, parasitosis, gastro-enteritis) or washing-water related illnesses (conjunctivitis and skin diseases)[14] represented 69% of all infections (fig. 1), suggesting a major role for water-borne diseases in the overall health status of the population. In view of this significant health burden posed by water-borne diseases in this population, an impact of the adequacy of the village water supply on the treatment characteristics of malnourished children managed in an ambulant fashion in the region’s therapeutic feeding centers seemed plausible.

Out of the 20 sample villages, 1518 moderately malnourished children (22% of all children in the MSF feeding program in the region) were enrolled in ambulatory treatment at one of the therapeutic feeding centers. Relatively long lengths of stay in the feeding program were observed among these children: overall, only 423 (28%) children spent 6 weeks or less in the program, while 467 (31%) were discharged from the program after more than 12 weeks. Typically, such programs aim to discharge children after no more than 6 weeks[9], [10]. Stratifying these children according to the adequacy of the water supply in their home village (neither quantity nor quality adequate; adequate quantity; adequate quality; and both adequate quantity and quality) revealed clear differences in the lengths of stay in the therapeutic feeding program. Of the children exposed to water inadequacy (i.e. inadequate quality, quantity, or quality and quantity), higher proportions fell into the longer stay categories (6–8 weeks, 8–12 weeks and more than 12 weeks) than of children residing in villages with adequate quantity and quality of the water supply (fig. 2).

Generalized Estimating Equation analysis with an ordinal logistic regression model was performed to assess the relative contributions of water quantity versus quality to the length of stay in the therapeutic feeding programs in this study setting. Distance of the villages to their respective therapeutic feeding centers (ranging from 0.3 to 40 km) was considered as a possible confounding factor but was found not to be associated with length of stay, and was therefore not included in subsequent models. Both adequate water quantity (Odds Ratio = 0.64, 95%Confidence intervals 0.46–0.90) and quality (OR = 0.44, 95%CI 0.32–0.58) were found to be independently associated with shorter lengths of stay in the feeding program, though adequate water quality seemed to carry a stronger association. Length of stay in the program was shortest in children from villages with both adequate water quality and quantity (OR = 0.37, 95%CI 0.23–0.58) (fig. 3).

## Discussion

This study demonstrates a strong correlation between the adequacy of the water supply in villages of malnourished children in an ambulatory feeding program and their length of stay in that program. This may be related to the observed association between adequacy of water supply and prevalence of secondary water-related infections. It supports the earlier informal program assessment that children in the therapeutic feeding program in this region of Niger were remaining longer than expected in the program, considering that the length of stay in similar programs elsewhere is typically six weeks[9], [10]. Additionally, the study indicates that adequate water quality and water quantity are independently associated with improved program outcomes, with a slightly higher impact of water quality over quantity. As such, we bring new evidence to the long-standing debate on the relative importance of water quality versus quantity[15], [16], [17], [18].

In Niger, malnutrition affects mainly children below two years of age and its causes are multiple: lack of adequate weaning food, unhygienic living conditions, poor access to health care and inadequate water supply. The high proportion of water-related infections in this study (almost 70%) reinforces the role of water as a contributing factor to malnutrition and delays in therapeutic response. Additionally, by demonstrating the correlation between adequacy of water sourcing and an increasing length of stay in the therapeutic program, this study highlights the critical role of access to adequate quality and quantity of water. A clear policy implication resulting from this study is that providing therapeutic food does not suffice. Ambulatory therapeutic feeding programs should include an assessment of the adequacy of the water supply, both at the level of quantity and quality, in the villages treatment of malnourished children is planned. A protocol for rapid and accurate field assessment of this should be developed and included in a feeding program’s plans. A question which will logically follow from such assessments is how to handle inadequacy of water supplies in an area afflicted by malnutrition? Each situation will naturally require a locally-adapted response, but the principles of providing water of adequate quality in adequate quantities are well-known[19].

This study suffered from considerable limitations. Due to operational constraints in the field, data on potential confounders such as socio-demographic factors, sanitation practices in the studied villages, mothers’ workloads, etc. was not accessible. Likewise, expanded data on water quality and quantity at the village level (number of water sources, quality of individual sources, frequency of use, sourced amounts, etc.) could not be obtained. Additionally, as a result of aggregating the data across 6 months, seasonal effects could not be assessed, though any bias resulting from studying only one season (wet or dry) was removed by collecting data for equal periods of time in the wet and the dry season. Stratification of the data per season could reveal whether the observed associations between water quality/quantity and length of stay in the therapeutic feeding program are linked to a specific season. As such information could help guide operational strategies of e.g. nutritional programs, seasonal variation should be included in future study initiatives. In terms of geospatial characteristics of the villages, some confounders were crudely corrected for by factoring the distance of the villages to the feeding centers into the analysis, we cannot exclude that location-associated characteristics factors such as frequency of visits to collect food also impacted on the length of stay in the program. Another limitation was the use of a proxy indicator for water quality (sourcing from a protected well) – while in line with studies performed by WHO and UNICEF[12], this definition could be refined by running biochemical and microbiological tests on the water itself.

In conclusion, this study suggests that outpatient therapeutic feeding programs need to assess the adequacy of water supply in villages where their patients live if they are to be effective in reducing malnutrition in a timely manner. The study underlines the importance of a holistic approach to nutritional support. More definitive studies on the relationship between water quantity and quality and childhood nutrition are needed.
